# Analysis of the structure and metabolic function of microbial community in cigar tobacco leaves in agricultural processing stage

**DOI:** 10.3389/fmicb.2023.1230547

**Published:** 2023-08-10

**Authors:** Qianying Zhang, Tianfei Zheng, Zhen Yang, Shuanghong Yang, Wen Cai, Pinhe Li, Yang Huang, Juan Zhang, Dongliang Li

**Affiliations:** ^1^Cigar Fermentation Technology Key Laboratory of China Tobacco, Cigar Technology Innovation Center of China Tobacco, China Tobacco Sichuan Industrial Co., Ltd., Chengdu, China; ^2^School of Biotechnology, Jiangnan University, Wuxi, China

**Keywords:** cigar tobacco leaf (CTL), air-curing, fermentation, microbial community, metabolic function

## Abstract

The agricultural fermentation processing of cigar tobacco leaves (CTLs), including air-curing and agricultural fermentation, carried out by tobacco farmers has rarely been studied. In this study, we have investigated the microbial community in the CTLs during air-curing and agricultural fermentation by 16S rRNA and ITS gene high-throughput sequencing. The results showed that the richness of microbial communities gradually increased with the development of agricultural fermentation, which means that not all microorganisms in CTLs come from the fields where tobacco grows, but gradually accumulate into CTLs during the fermentation process. *Enterobacteriaceae*, *Chloroplast*, and *Alternaria* were the dominant genera in the air-cured CTLs. *Aquabacterium*, unclassified *Burkholderiaceae*, *Caulobacter*, *Brevundimonas*, and *Aspergillus* were the dominant genera in the agriculturally fermented CTLs. *Acinetobacter*, *Methylobacterium*, *Sampaiozyma*, and *Plectosphaerella* first significantly increased, and then significantly decreased during agricultural processing. The changes in microbial communities are mainly related to their different functions during fermentation. This means that when the fermentation effect of the original microbial community in cigar tobacco leaves is not ideal, we can optimize or design the microbial community based on the fermentation function that the microbial community needs to achieve. These results may help adjust and optimize the agricultural fermentation process of CTLs, and help develop the quality of CTLs and increase the income of tobacco farmers.

## 1. Introduction

Tobacco (*Nicotiana tabacum* L.) is the most widely cultivated non-food crop in the world (Banožić et al., 2020). Cigar is a tobacco product that has been dried and fermented. Fresh cigar tobacco leaves (CTLs) after harvest usually require air-curing and agricultural fermentation performed by farmers before they can be sold to industrial companies. Air-curing is done to remove moisture from CTLs and change the color of CTLs from green to yellow. The most important thing is that the macromolecular substances in CTLs (starch, cellulose, proteins, etc.) begin to degrade into reducing sugars and amino acids. Agricultural fermentation can be considered as a natural continuation of air-curing, where macromolecular substances continue to degrade and reducing sugars and amino acids begin to be converted into various aromatic substances. Air-curing and agricultural fermentation are important agricultural processes that determine the quality of cigar leaves (Jin, 1982; Toharisman et al., 2008). However, relying solely on experience in agricultural fermentation can easily cause uneven quality of tobacco leaves from different batches.

Microbes have been found to play an important role in the fermentation of CTLs (Reid et al., 1944). Based on culture-independent molecular biology techniques such as polymerase chain reaction denaturing gradient gel electrophoresis (PCR-DGGE) (Giacomo et al., 2007) and Illumina MiSeq sequencing (Zhang et al., 2018a,b) have been used to characterize microbial communities associated with CTLs, *Bacillus*, *Staphylococcus*, *Penicillium*, *Debaryomyces*, *Jeotgalicoccus*, *Lactobacillus*, *Weissella*, *Yania*, *Pseudomonas*, and *Acinetobacter* have been identified in many tobacco samples (Li et al., 2009; Du et al., 2016; Zhang et al., 2018a,b; Liu et al., 2021). However, few studies have tracked the succession of structure and function of microbial communities in CTLs during agricultural processing stage.

In this study, we investigated the structure and function of microbial communities in CTLs during the agricultural processing by 16S rRNA and ITS gene high-throughput sequencing. Exploring the dynamic change of microbial community during agricultural processing stage would be helpful to master the change pattern of microbial communities in CTLs, and investigating the function of microbial communities would be helpful to understand the mechanism of microbial community on CTLs. These results may have important contributions to improving the quality of Chinese CTLs.

## 2. Materials and methods

### 2.1. CTLs sampling

The CTLs investigated in this study were Dexue CTLs cultivated in Shifang, Sichuan Province, China. Shifang is known as the cigar production base in China, with a history of nearly 400 years of cigar tobacco planting and cigar production. The agricultural processed CTLs, including freshly harvested, air-cured, and agricultural fermented CTLs were collected, 500–1,000 g each sample, then CTLs were transferred into sterile bags, and stored at −20°C until further study. All CTLs were performed in triplicate. Freshly harvested, air-cured, and agriculturally fermented Dexue Nos.1, 3, 4, and 7 CTLs were marked accordingly (D1X, D3X, D4X, D7X, D1C, D3C, D4C, D7C, D1F, D3F, D4F, and D7F).

### 2.2. DNA extraction and Illumina MiSeq sequencing

The CTLs (5.0 g) was suspended in 100 mL of sterile PBS and shaken for 2 h at 200 rpm, after which the supernatant was centrifuged at 10,000× *g* for 30 min. Total genomic DNA of each sample was extracted using an EZNA® Soil DNA Kit (Omega, USA) according to the manufacturer’s instructions. The genomic DNA was amplified and sequenced using the V4–V5 hypervariable region of 16S rRNA genes (forward primer 515F: 5’-GTGCCAGCMGCCGCGGTAA-3′; and reverse primer 907R: 5’-CCGTCAATTCMTTTRAGT TT-3′) (Parada et al., 2016) and the ITS1 hypervariable region of internal transcribed spacer (ITS) genes (forward primer ITS1F: 5’-CTT GGT CAT TTA GAG GAA GTA A-3′; and reverse primer ITS2R: 5’-GCT GCG TTC TTC ATC GAT GC-3′) (Usyk et al., 2017).

Deoxyribonucleic acid libraries were validated by TruSeq Nano DNA LT (Illumina, USA) and quantified using a PicoGreen dsDNA Assay Kit (Invitrogen, USA). Amplicons were pooled in equal amounts, and sequencing was performed using a 2 × 300 paired-end (PE) configuration using an Illumina MiSeq sequencing system according to the manufacturer’s instructions (Illumina, USA). Operational taxonomic units (OTUs) of qualified sequences were identified using the clustering program VSEARCH version 1.9.6 and the SILVA version 132 database (Rognes et al., 2016) with 97% similarity. The alpha diversity, including the Chao1 and Shannon values, was analyzed using QIIME version 1.9.1 (Caporaso et al., 2010).

### 2.3. Data analysis

Significant differences among the freshly harvested, air-cured, and agricultural fermented CTLs groups were determined using SPSS version 19 (IBM, USA), by one-way analysis of variance (ANOVA) and Duncan’s multiple comparison test (*p* < 0.05). In order to further investigate the changes in microbial abundance, circular visualization diagrams were used to display the overlapping and differentiating microbial taxa among the three courses of agricultural processing of CTLs at the phylum and genus levels using Circos software online (http://circos.ca; Krzywinski et al., 2009). Phylogenetic Investigation of Communities by Reconstruction of Unobserved States (PICRUSt2) and Fungi Functional Guild (FUNGuild) were used to predict the metagenomic functions based on the normalized OTU tables (Douglas et al., 2019), which were compared using Statistical Analysis of Metagenomic Profiles (STAMP) version 2.1.3 (Parks et al., 2014). Partial least-squares regression (PLS) was conducted using SIMCA-P (version 13.0; UMETRICS, Sweden) to construct statistical models of bacteria and fungi for correlation between microbial genera (X) and functional genes (Y). The data are presented as means, which were scaled before the analysis. In the PLS model, the variable importance for the projection (VIP) reflects the importance of the variables both to explain X and to correlate to Y. Variable importance for the projection values exceeding 1 indicated the ‘important’ X-variables. The PLS regression coefficients (CoeffCS) are coefficients used for interpreting how strongly Y is correlated with the systematic part of each of the X-variables. Networks were explored and visualized using the interactive platform Gephi (Bastian et al., 2009) based on the CoeffCS of the VIPs, with each node representing one genus and one functional gene and edges representing a strong and significant correlation.

## 3. Results

### 3.1. Microbial community diversity

In total, 1,741,054 and 2,095,371 reads of the 16S rRNA and ITS gene sequences were obtained from all CTLs. Table 1 shows the diversity indices of the bacterial and fungal communities in the CTLs. The coverage was more than 99%, indicating that Illumina MiSeq sequencing was deep enough to represent all the detected microbial communities. The Chao1 values of the bacterial communities of the air-cured and agriculturally fermented CTLs were higher than those of the freshly harvested CTLs (*p* < 0.05). The Shannon values of the microbes in the air-cured CTLs were higher than those in the freshly harvested and agriculturally fermented CTLs (*p* < 0.05). These results showed that the diversity of microbial communities gradually decreased with the progress of agricultural fermentation, which means that not all microorganisms in CTLs come from the fields where tobacco grows, but gradually accumulate into CTLs during the fermentation process.

**Table 1:** Diversity indices of the bacterial and fungal communities in the cigar tobacco leaves (CTLs).

Sample	Bacterial diversity			Fungal diversity		
	Chao1	Shannon	Coverage	Chao1	Shannon	Coverage
D1X	236.83	3.41	0.997	219.25	3.10	0.999
D3X	479.34	4.62	0.993	171.91	2.86	0.998
D4X	322.07	3.74	0.996	186.68	1.84	0.998
D7X	362.49	3.80	0.999	262.66	3.11	0.998
D1C	872.47	6.95	0.991	122.42	3.19	0.999
D3C	1091.63	6.72	0.996	162.87	3.57	0.999
D4C	620.55	5.89	0.993	125.62	2.91	0.999
D7C	397.59	4.95	0.996	122.31	3.34	0.999
D1F	546.13	4.93	0.994	25.17	0.16	0.999
D3F	514.86	5.09	0.995	117.51	1.30	0.999
D4F	421.45	4.55	0.996	50.76	0.92	0.999
D7F	358.32	5.02	0.997	107.64	1.97	0.999

D1X to D7X denote the freshly harvested CTLs, D1C to D7C denote the air-cured CTLs, and D1F to D7F denote the agriculturally fermented CTLs.

### 3.2. Microbial community composition and structure

**Figure 1:**
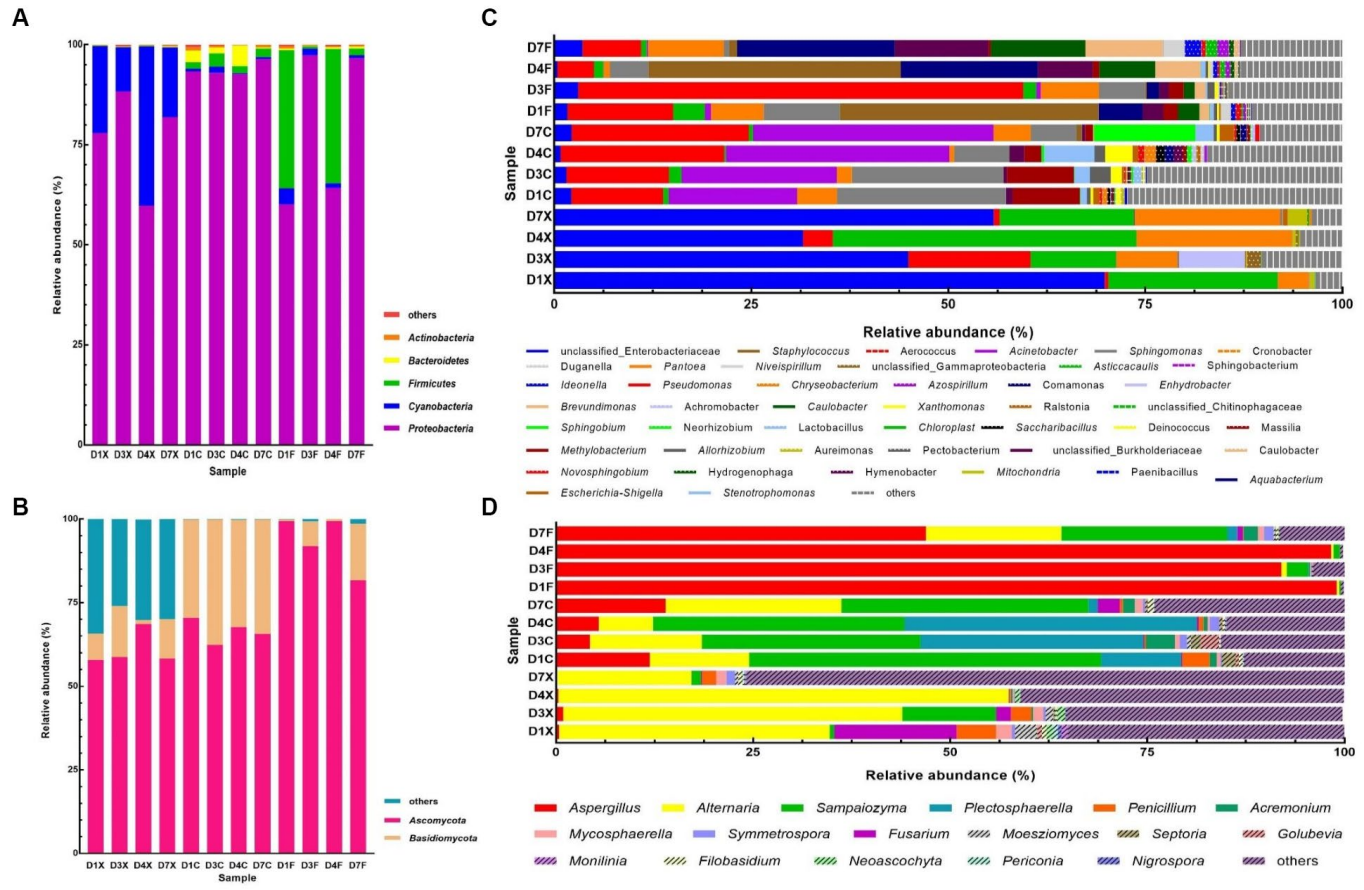
Plot of the phylum and genus levels’ relative abundances for the bacterial and fungal communities in the cigar tobacco leaves (CTLs). Panels **(A,C)** represent the bacterial communities at the phylum and genus levels. Panels **(B,D)** represent the fungal communities at the phylum and genus levels. D1X to D7X denote the freshly harvested CTLs, D1C to D7C denote the air-cured CTLs, and D1F to D7F denote the agriculturally fermented CTLs.

Taxonomic analysis of the reads revealed that *Proteobacteria*, *Cyanobacteria*, *Firmicutes*, *Bacteroidetes, Actinobacteria*, *Ascomycota* and *Basidiomycota* were dominant at the phylum level (Figures 1A,B). A total of 610 bacterial genera and 473 fungal genera were detected, of which 45 and 17 had a relative abundance higher than 0.5% in at least one sample (Figures 1C,D). The dominant bacterial genera, with relative abundance higher than 5.0% in at least one sample, were unclassified *Enterobacteriaceae*, *Pseudomonas*, *Chloroplast*, *Acinetobacter*, *Pantoea*, *Sphingomonas*, *Staphylococcus*, *Aquabacterium*, unclassified *Burkholderiaceae*, *Methylobacterium*, *Caulobacter*, and *Brevundimonas*, whose abundance accounts for 61.81–95.81% in all CTLs. The dominant fungal genera were *Aspergillus*, *Alternaria*, *Sampaiozyma*, and *Plectosphaerella*.

**Figure 2:**
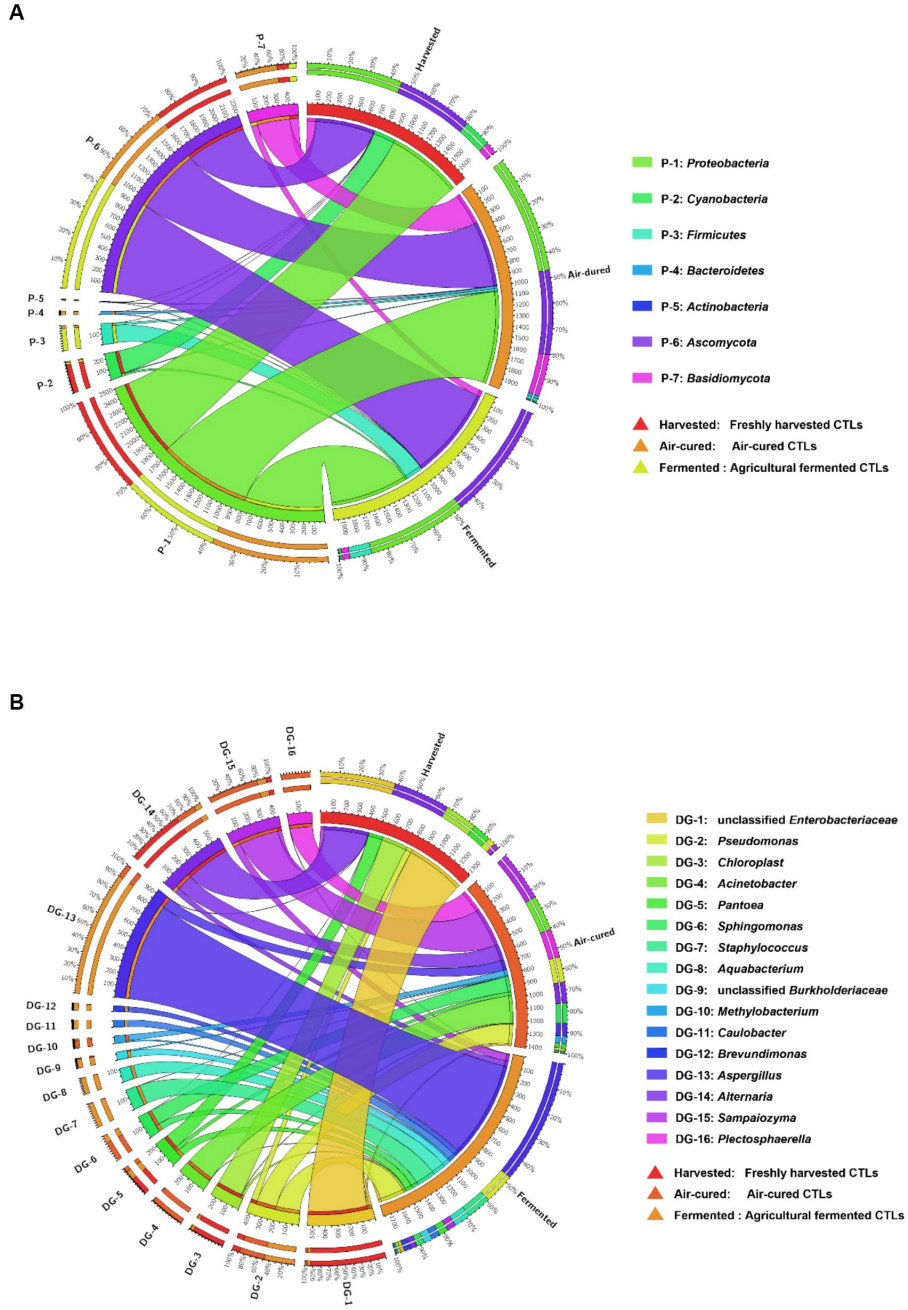
Differences in the microbial community composition among the three groups (harvested: freshly harvested cigar tobacco leaves [CTLs]; air-cured: air-cured CTLs; and fermented: agricultural fermented CTLs) at the phylum and genus level. Panels **(A,B)** represent the relative abundances of the phyla and genus shown in circular visualization (Circos). The thickness of each ribbon represents the abundance of each taxon. The absolute tick above the inner segment and relative tick above the outer segment indicate the read and relative abundances of each taxon, respectively.

Differences in the dominant phyla and genera in the different courses of agricultural processing of all samples were shown in the Circos diagrams (Figures 2A,B, respectively), which clearly showed that the dominant phyla and genera were more diverse in the air-cured and agriculturally fermented CTLs than in those in the freshly harvested CTLs. At phylum level, *Cyanobacteria* were mainly present in the freshly harvested CTLs (D1X, D3X, D4X, and D7X), *Bacteroidetes* and *Basidiomycota* were mainly present in the air-cured CTLs (D1C, D3C, D4C, and D7C), and *Ascomycota* was mainly present in the agriculturally fermented CTLs (D1F, D3F, D4F, and D7F) (*p* < 0.05). At genus level, unclassified *Enterobacteriaceae*, *Chloroplast*, *Pantoea*, and *Alternaria* were the dominant genera in the air-cured CTLs. The *Acinetobacter*, *Sphingomonas, Methylobacterium*, *Sampaiozyma*, and *Plectosphaerella* increased first and then decreased during agricultural processing. *Pseudomonas*, *Staphylococcus*, *Aquabacterium*, unclassified *Burkholderiaceae*, *Caulobacter*, *Brevundimonas*, and *Aspergillus* were the dominant genera in the agriculturally fermented CTLs.

### 3.3. Metabolic function prediction of microbial communities

**Figure 3:**
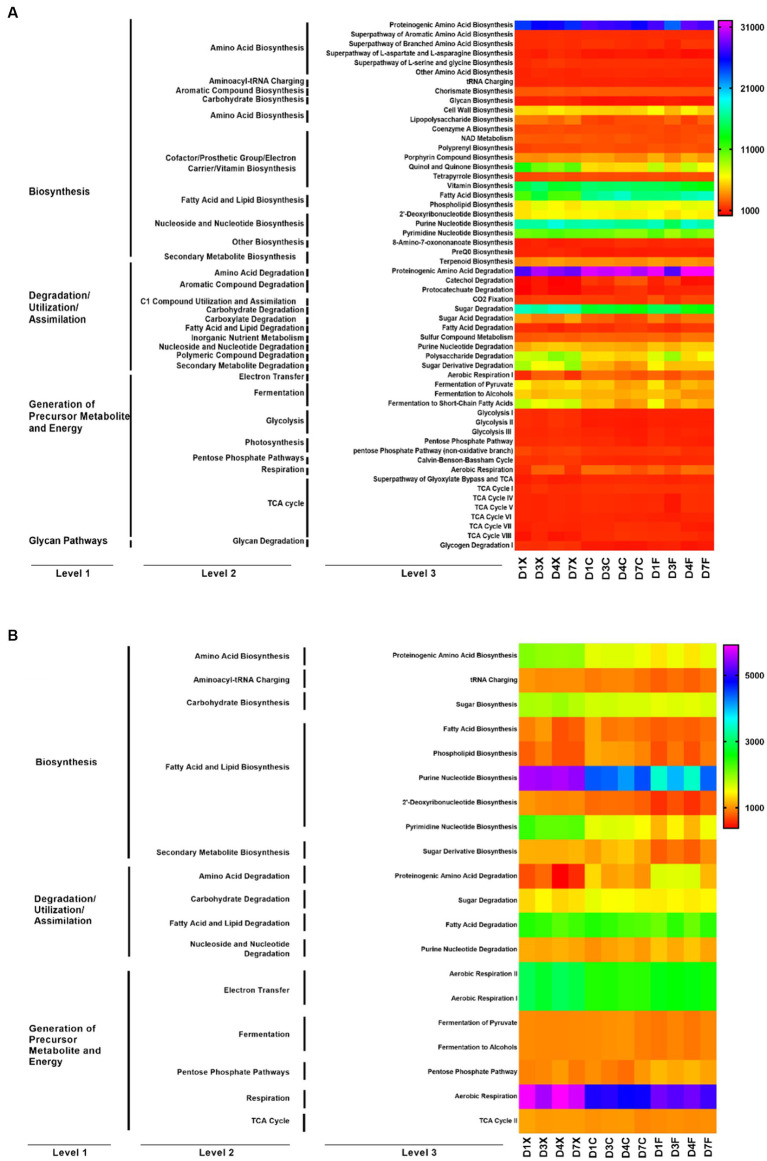
Function prediction of the bacteria **(A)** and fungi **(B)** in the cigar tobacco leaves (CTLs). D1X to D7X denote the freshly harvested CTLs, D1C to D7C denote the air-cured CTLs, and D1F to D7F denote the agriculturally fermented CTLs.

The metabolic function of microbial communities was predicted using PICRUSt2 and FUNGuild (Figure 3). Functions of the bacteria and fungi in the CTLs involved fatty acid and lipid biosynthesis, amino acid biosynthesis, fermentation, and carbohydrate biosynthesis. The abundance of 56 bacterial functions and 20 fungal functions were higher than 1,000. Bacteria are mainly involved in amino acid biosynthesis, fatty acid and lipid biosynthesis, nucleoside and nucleotide biosynthesis, amino acid degradation, and carbohydrate degradation at Kyoto Encyclopedia of Genes and Genomes (KEGG) level 2, including fatty acid biosynthesis, proteinogenic amino acid biosynthesis, purine nucleotide biosynthesis, proteinogenic amino acid degradation, and sugar degradation at level 3. Fungi are mainly involved in fatty acid and lipid biosynthesis, fatty acid and lipid degradation, electron transfer, and respiration at KEGG level 2, including purine nucleotide biosynthesis, pyrimidine nucleotide biosynthesis, fatty acid degradation, aerobic respiration II, aerobic respiration I, and aerobic respiration at level 3.

**Figure 4:**
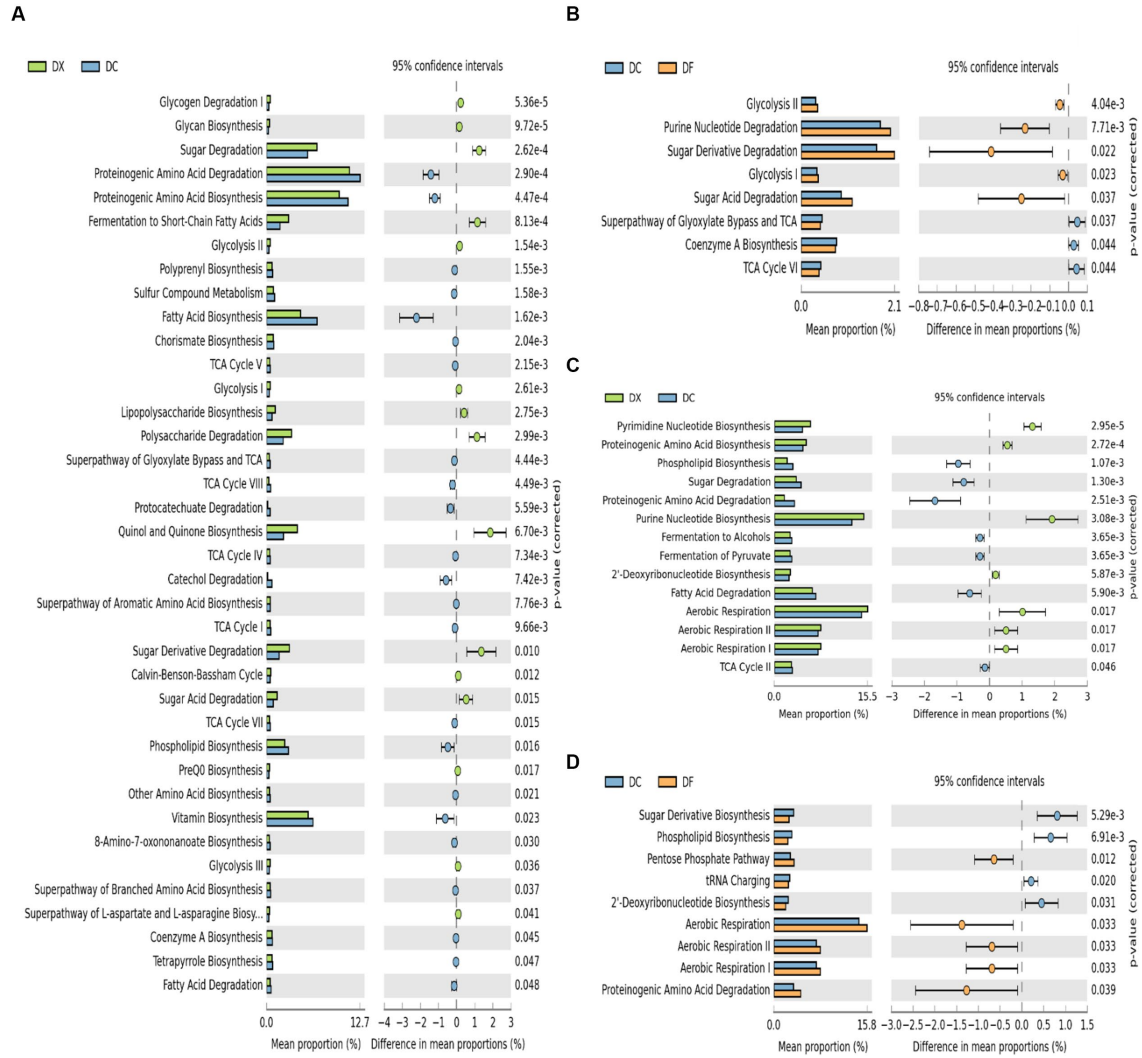
Differences in the microbial function composition among the three groups (DX: freshly harvested cigar tobacco leaves [CTLs]; DC: air-cured CTLs; DF: agriculturally fermented CTLs). Panels **(A–D)** represent the Statistical Analysis of Metagenomic Profiles (STAMP) analysis of the relative abundance of the bacterial and fungal functions. *p* < 0.05 indicates significant differences between two different site types. Error bars represent Welch’s t-interval.

Significant differences in the functional prediction profiles of the CTLs at different agricultural processing among the three courses were observed in KEGG level 3 (Figure 4). For the bacterial communities, a total of 38 out of 56 (41.01% increased, 28.57% decreased) were significantly different between the freshly harvested and air-cured CTLs, and eight out of 56 (8.93% increased, 5.36% decreased) were significantly different between the air-cured and fermented CTLs (Figures 4A,B). For the fungal communities, a total of 14 out of 20 (35% increased, 35% decreased) were significantly different between the freshly harvested and air-cured CTLs, and nine out of 20 (25% increased, 20% decreased) were significantly different between the air-cured and fermented CTLs (Figures 4C,D).

### 3.4. Correlation between microbial genera and functional genes

**Figure 5:**
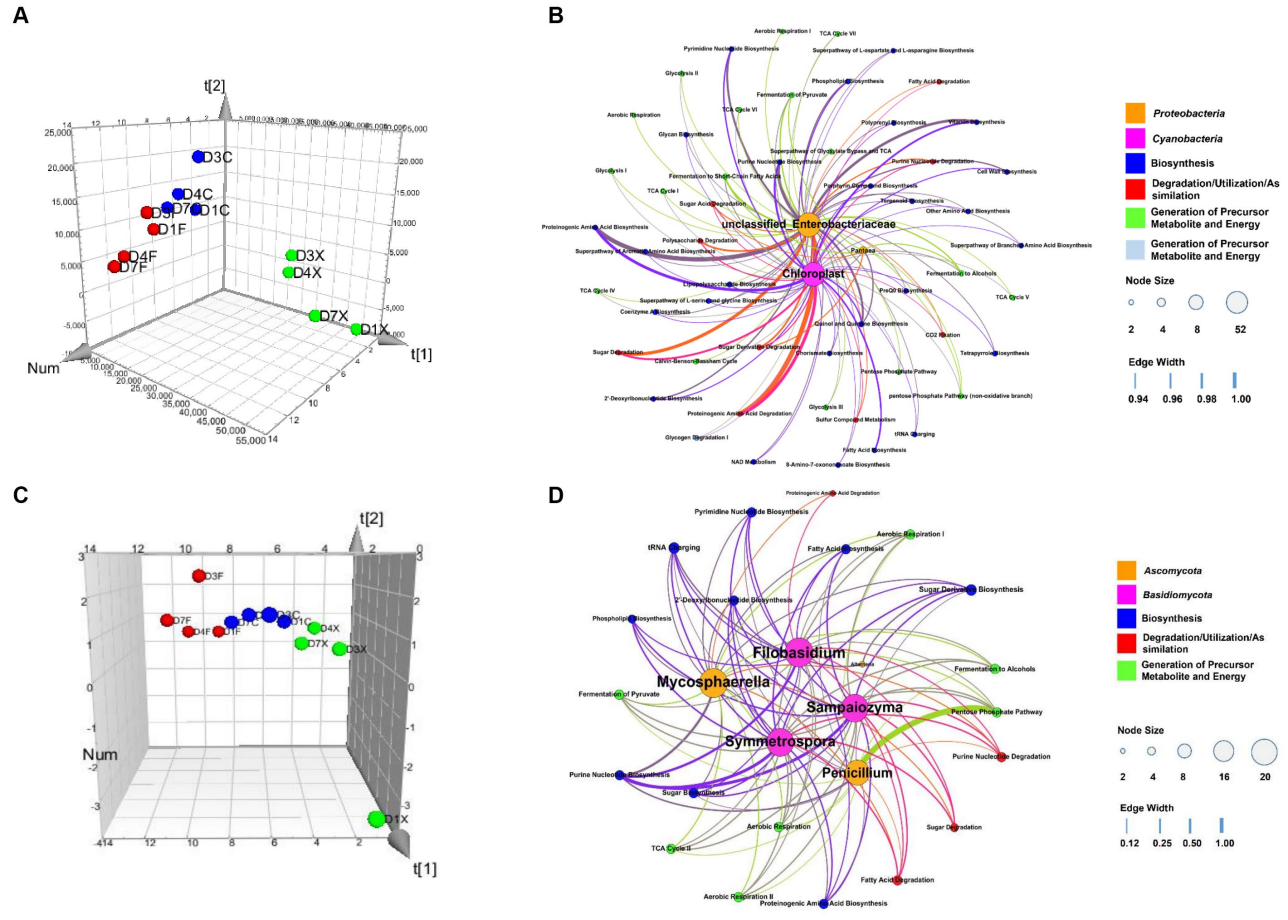
Score scatter three-dimensional plots of the partial least-squares regression (PLS) **(A,C)** and network of correlation between the microbial genera and functional genes **(B,D)** based on the PLS. The PLS of the bacterial **(A)**, fungal **(C)**, and various CTLs is represented as a two-dimensional representation of the scores (t[1] and t[2]) on the first and second PLS components. Relationship between the functional communities and bacteria **(B)** and fungi **(D)**, with significant changes in abundance. For each panel, the size of each node is proportional to the number of connections, nodes of the same color are affiliated with the same genus or function, and the thickness of each connection between two nodes is proportional to the value of the PLS regression coefficients (CoeffCS) with statistical significance (*p* < 0.05). D1X to D7X denote the freshly harvested CTLs, D1C to D7C denote the air-cured CTLs, and D1F to D7F denote the agriculturally fermented CTLs.

A model based on Partial least-squares regression was used tialto analyze the correlation between microbial genera and functional genes. And two significant principal components of the total variance in the data matrix were extracted in both the bacterial and fungal models. For the bacterial model, the R2X, R2Y, and Q2 were 0.886, 0.871, and 0.809, respectively, which meant that 88.6% variation was due to these two components, with a total of 87.1% dummy Y variable per class, and 80.9% overall cross-validated R2 for these two components. The data indicated that the PLS model was suitable for this study. Samples of the freshly harvested, air-cured, and fermented cigar leaves were clearly separated on the score scatter plot (Figure 5A, Table 2), with the freshly harvested group located on the right side of the plot, the air-cured group located on the upper left side of the plot, and the fermented group located on the left-middle side of the plot. The VIP values were unclassified *Enterobacteriaceae*, *Pseudomonas*, *Chloroplast*, *Acinetobacter*, *Pantoea*, and *Sphingomonas* in the first and second significant principal components (Table 3). The coefficients refer to the PLS model being rewritten as a regression model. The correlation network between the VIP genera and functional genes was described by significant CoeffCS, as shown in Figure 5B. There were 52 and 51 edges (functions) significantly positively correlated with unclassified *Enterobacteriaceae* and *Chloroplast*, and the top five functions of both were proteinogenic amino acid degradation, proteinogenic amino acid biosynthesis, sugar degradation, purine nucleotide biosynthesis, and vitamin biosynthesis. Eight functions were significantly positively correlated with *Pantoea*, and the top five functions were lipopolysaccharide biosynthesis, quinol and quinone biosynthesis, sugar acid degradation, sugar derivative degradation, and fermentation of pyruvate.

**Table 2:** Coefficient values between the variables and groups.^†^

Variable	CoeffCS[1]			CoeffCS[2]		
	DA(1)	DA(2)	DA(3)	DA(1)	DA(2)	DA(3)
Unclassified *Enterobacteriaceae*	0.5778*	0.1442	−0.7220*	1.6971*	−1.4545*	−0.2426
*Pseudomonas*	−0.1946	−0.0485	0.2431	−0.4259*	0.2819	0.1440
*Chloroplast*	0.2374	0.0592	−0.2966*	0.7340*	−0.6501*	−0.0839
*Acinetobacter*	0.0652	0.0163	−0.0815	−0.7220*	1.1407*	−0.4187*
*Pantoea*	0.0672	0.0168	−0.0840	0.3130*	−0.3342*	0.0212
*Sphingomonas*	−0.0380	−0.0095	0.0475	−0.4258*	0.5444*	−0.1186
*Staphylococcus*	−0.2465*	−0.0615	0.3080	−0.0731	−0.30928*	0.3823*
*Aquabacterium*	−0.1664*	−0.0415	0.2080	−0.0512	−0.2061	0.2573
Unclassified *Burkholderiaceae*	−0.0826*	−0.0206	0.1032	−0.0530*	−0.0629	0.1159
*Methylobacterium*	−0.0030	−0.0007	0.0038	−0.1581*	0.2208*	−0.0627
*Caulobacter*	−0.0868*	−0.0216	0.1084	−0.0267	−0.1075	0.1341
*Brevundimonas*	−0.0680*	−0.0170	0.0849	−0.0222	−0.0823	0.1045
*Stenotrophomonas*	0.0039	0.0010	−0.0049	−0.0869*	0.1307*	−0.0438
*Allorhizobium*	−0.0028	−0.0007	0.0034	−0.0389*	0.0509*	−0.0120
*Xanthomonas*	0.0008	0.0002	−0.0010	−0.0435*	0.0635*	−0.0200
*Mitochondria*	0.0106	0.0026	−0.0132	0.0311	−0.0267*	−0.0044
*Novosphingobium*	−0.0052*	−0.0013	0.0065*	−0.0146*	0.0122*	0.0024
*Azospirillum*	−0.0090*	−0.0022	0.0112	−0.0028	−0.0110	0.0139
*Chryseobacterium*	0.0015	0.0004	−0.0019	−0.0177*	0.0278*	−0.0101
*Saccharibacillus*	0.0011	0.0003	−0.0014	−0.0166*	0.0255*	−0.0089
*Comamonas*	0.0014	0.0003	−0.0017	−0.0141	0.0225*	−0.0084*
*Massilia*	0.0005	0.0001	−0.0006	−0.0103*	0.0156*	−0.0052*
*Hydrogenophaga*	−0.0046*	−0.0011	0.0057	−0.0014	−0.0057	0.0071
*Neorhizobium*	0.0007	0.0002	−0.0009	−0.0086*	0.0135*	−0.0049
*Achromobacter*	0.0008	0.0002	−0.0009	−0.0081	0.0128*	−0.0047*
*Pectobacterium*	−0.0032*	−0.0008	0.0041*	−0.0018*	−0.0029*	0.0047*
Unclassified *Chitinophagaceae*	0.0015	0.0004	−0.0018*	0.0043*	−0.0036*	−0.0006
*Aspergillus*	−1.2266*	−0.3060	1.5326*	−0.6147*	−1.1800*	1.7947*
*Alternaria*	0.4261*	0.1063	−0.5324*	0.8697*	−0.5273	−0.3424*
*Sampaiozyma*	0.0502	0.0125	−0.0627	−0.9408*	1.4279*	−0.4871*
*Plectosphaerella*	0.0523	0.0130	−0.0653	−0.5851*	0.9234*	−0.3383
*Penicillium*	0.0318*	0.0079	−0.0397*	0.0481	−0.0153	−0.0328*
*Mycosphaerella*	0.0132*	0.0033	−0.0164*	0.0238*	−0.0119	−0.0119*
*Moesziomyces*	0.0124	0.0031	−0.0155*	0.0315	−0.0241	−0.0074*
*Septoria*	0.0036	0.0009	−0.0044	−0.0267*	0.0441*	−0.0174
*Filobasidium*	0.0039*	0.0010	−0.0049*	0.0013	0.0047	−0.0060*
*Neoascochyta*	0.0073	0.0018	−0.0091*	0.0200*	−0.0164*	−0.0036
*Periconia*	0.0028	0.0007	−0.0035	0.0060	−0.0038	−0.0021*
*Nigrospora*	0.0024	0.0006	−0.0030*	0.0048	−0.0028	−0.0020*

^†^CoeffCS [1] and CoeffCS [2] represent the significant principal components 1 and 2, respectively. DA (1), freshly harvested CTL group; DA, (2) air-cured CTL group; and DA, (3) agriculturally fermented CTL group. ^*^Coefficient is significant (above noise). CoeffCS, partial least-squares regression coefficients.

**Table 3:** Variable importance for the projection (VIP) values of the variables to the first and second principal components.^†^

Variable	Bacterial model		Variable	Fungal model	
	VIP[1]	VIP[2]		VIP[1]	VIP[2]
Unclassified *Enterobacteriaceae*	5.27885*	4.46401*	*Septoria*	1.00788*	0.94554
*Pseudomonas*	2.18363*	3.01881*	*Filobasidium*	1.22234*	1.13426*
*Chloroplast*	2.34807*	1.82644*	*Alternaria*	1.22596*	1.17350*
*Acinetobacter*	1.39447	2.24928*	*Sampaiozyma*	1.22290*	1.20344*
*Pantoea*	1.65344*	1.27616*	*Penicillium*	1.05662*	1.01789*
*Sphingomonas*	1.08605	1.74126*	*Mycosphaerella*	1.29008*	1.19901*
			*Symmetrospora*	1.31005*	1.25677*

^†^VIP [1] and VIP [2] represent the significant principal components 1 and 2, respectively. ^*^Coefficient is significant (above noise).

For the fungal model, the R2X, R2Y, and Q2 were 0.680, 0.826, and 0.760, respectively, which meant that 68.0% of the variation was due to these two components, with a total of 82.6% dummy Y variable per class and 76.0% overall cross-validated R2 for these two components. The data indicate that the PLS model was suitable for this study. Samples of the freshly harvested, air-cured, and fermented cigar leaves were clearly separated on the score scatter plot (Figure 5C, Table 2), and the freshly harvested group was located on the right side of the plot, the air-cured group was located on the middle side of the plot, and the fermented group was located on the left side of the plot. The VIP values were *Septoria*, *Filobasidium*, *Alternaria*, *Sampaiozyma*, *Penicillium*, *Mycosphaerella*, and *Symmetrospora* in the first and second principal components (Table 3). The correlation network is shown in Figure 5D. The tRNA charging and sugar derivative biosynthesis were significantly and positively correlated with *Alternaria*. Twenty functions were significantly positively correlated with *Sampaiozyma* and *Symmetrospora*, and the top five functions of both were fatty acid biosynthesis, phospholipid biosynthesis, sugar derivative biosynthesis, sugar degradation, fermentation of pyruvate, and fermentation to alcohols. There were 16 and 21 functions that were significantly positively correlated with *Penicillium* and *Mycosphaerella*, and the top five functions of both were proteinogenic amino acid biosynthesis, 2′-deoxyribonucleotide biosynthesis, pyrimidine nucleotide biosynthesis, purine nucleotide biosynthesis, and sugar derivative biosynthesis. Phospholipid biosynthesis, 2′-deoxyribonucleotide biosynthesis, pyrimidine nucleotide biosynthesis, purine nucleotide biosynthesis, and sugar derivative biosynthesis were the top five functions significantly positively correlated with *Filobasidium*.

## 4. Discussion

Air-curing and agricultural fermentation are important production processes that determine the quality of CTLs and finished products (Zhang et al., 2020a,b). In this study, the structure and diversity of microbial communities in CTLs at different stages of agricultural processing in Shifang, Sichuan were studied by using the Illumina miseq sequencing method based on 16S rRNA and ITS genes. The results indicated that there are significant differences in the microbial communities of CTLs at different stages of agricultural processing, and the differences in the microbial communities during different stages of agricultural processing are even greater than those between different varieties. Our research helps to grasp the trend of microbial community changes in the agricultural fermentation process of CTLs, reveal the essence of CTL fermentation, and is of great significance for producing stable quality CTLs. In addition, understanding the succession and function of microbial community helps to reasonably regulate the microbial community to produce higher quality tobacco leaves.

At the phylum level, *Proteobacteria*, *Cyanobacteria*, *Firmicutes*, *Bacteroidetes, Actinobacteria*, *Ascomycota*, and *Basidiomycota* were abundant in the CTLs, which is similar to the results of previous studies based on flue-cured tobacco leaves (Zhang et al., 2020a,b). Except for *Cyanobacteria*, the microbial phyla in the cigar products were associated with the results obtained in this study (Ye et al., 2021). The abundance of *Cyanobacteria* in the freshly harvested CTLs was higher than those in the air-cured and agricultural fermented. *Cyanobacteria* have plant-type photosynthetic organs, which are mainly used to fix carbon dioxide through the reduction of pentose phosphate (Stal, 2015). The most dominant bacterial phylum was *Proteobacteria*, and the most dominant fungal phylum was *Ascomycota* in the CTLs from the Shifang, Sichuan province, which was consistent with those of the H382 cigar leaves from the Hainan province (Liu et al., 2021). At the genus level, there were 16 dominant genera, of which unclassified *Enterobacteriaceae*, *Pseudomonas*, *Acinetobacter*, *Pantoea*, *Sphingomonas*, *Staphylococcus*, and *Methylobacterium* were also the dominant bacterial genera in Mexican CTLs (Zhang et al., 2018a,b) and Hainan CTLs (Zhang et al., 2021).

The dominant genera were unclassified *Enterobacteriaceae*, *Chloroplast*, *Pantoea*, and *Alternaria* in the freshly harvested CTLs. The unclassified *Enterobacteriaceae* in the CTLs may be from the soil (Akita et al., 2019). *Chloroplast* are oxygen-producing phototrophic microorganisms that can form symbiotic relationships with plants and usually provide nitrogen fixation to plants (Rai et al., 2000). *Alternaria* can decompose cellulose in CTLs (Macris, 1984). After air-curing, the dominant microbes were *Acinetobacter*, *Sphingomonas, Methylobacterium*, *Sampaiozyma*, and *Plectosphaerella*. These microbes were reported to participate in the degradation of nicotine (Wang et al., 2011), lignin (Masai et al., 1999), and one-carbon compounds such as methanol and methylamine (Rossetto et al., 2011). After fermentation, the dominant microbes were *Pseudomonas*, *Staphylococcus*, *Aquabacterium*, unclassified *Burkholderiaceae*, *Caulobacter*, *Brevundimonas*, and *Aspergillus*. These microbes are mainly involved in the degradation of nicotine (Tang et al., 2009; Li et al., 2010), pectin, protein, starch (Schuster et al., 2002), xyloside, and oligosaccharides (Coenye, 2014), as well as the production of amino acids and fatty acids (Aro et al., 2010; Tabanelli et al., 2012) to form a series of flavoring substances and carbohydrates, thus improving the quality of CTLs. Although *Aspergillus* could improve the quality of cigar leaves by degrading nicotine or producing organic acids such as citric acid, it could cause mildew in cigar leaves (Yan et al., 2018). These results indicate that the microbial community in CTLs is not entirely derived from the soil where the tobacco plants are grown, but gradually recruited from the external environment during the fermentation process. The shifts in community structure and composition mainly follow the changes in CTLs, originating from changes in their metabolic function (Zhou et al., 2020; Chen et al., 2021).

Microorganisms are mainly involved in carbohydrate degradation, and biosynthesis of fatty acids, amino acids, and aroma components. Functional genes of fatty acid and lipid biosynthesis, aromatic compound degradation, and amino acid biosynthesis had higher relative abundances in the freshly harvested CTLs than in the air-cured and fermented CTLs, which meant microbes could synthesize fatty acids and amino acids and metabolize aromatic compounds in order to improve the flavor and aroma of CTLs (Wang, 2003). PLS is particularly useful when we need to predict a set of dependent variables from a large set of independent variables (Abdi, 2010). In this study, we used PLS to analyze the correlation between microbial genera and functional genes. PLS indicated that samples could be separated into three groups according to the processing process, which is consistent with the sampling results. Bacteria, mainly including unclassified *Enterobacteriaceae*, *Chloroplast*, and *Pantoea*, biosynthesized quinol and quinone in the freshly harvested CTLs the highest, which might promote the color change in the CTLs. Polyphenolic compounds in the CTLs can form brown pigments such as quinones through an enzymatic browning reaction in the air-curing process to make CTLs turn dark, thus affecting the color and aroma of the tobacco leaves (Guo et al., 2009). These bacteria also contributed to the degradation of total and reducing sugars through sugar degradation, sugar acid degradation, polysaccharide degradation, and sugar derivative degradation, and *Alternaria* contributed to sugar derivative biosynthesis, resulting in lower contents of total sugar and reducing sugar of the air-cured CTLs compared with those of freshly harvested CTLs (Wang, 2013).

In summary, our study systematically investigated the microbial community in CTLs during agricultural processing stage. The microbial communities vary widely due to fermentation time differences. The richness of microbial communities gradually increased with the development of agricultural fermentation. The changes in microbial communities are mainly related to their different functions during fermentation. This means that when the fermentation effect of the original microbial community in cigar tobacco leaves is not ideal, we can optimize or design the microbial community based on the fermentation function that the microbial community needs to achieve. These results may help adjust and optimize the agricultural fermentation process of CTLs, and help develop the quality of CTLs and increase the income of tobacco farmers.

## Data availability statement

The datasets presented in this study can be found in online repositories. The names of the repository/repositories and accession number(s) can be found in the article/supplementary material.

## Author contributions

DL and JZ conceived and designed the manuscript. QZ, TZ, ZY, SY, WC, PL, and YH handled samples and conducted experiments. QZ and TZ wrote and revised the manuscript. All authors read and approved the manuscript.

## Funding

This work was funded by China National Tobacco Company [Nos. 110202101062(XJ-11) and 110202201032(XJ-03)] and China Tobacco Sichuan Industrial Co., Ltd. (No. rtx201820).

## Conflict of interest

QZ, DL, ZY, SY, WC, PL, and YH were employed by China Tobacco Sichuan Industrial Co., Ltd.

The remaining authors declare that the research was conducted in the absence of any commercial or financial relationships that could be construed as a potential conflict of interest.

## Publisher’s note

All claims expressed in this article are solely those of the authors and do not necessarily represent those of their affiliated organizations, or those of the publisher, the editors and the reviewers. Any product that may be evaluated in this article, or claim that may be made by its manufacturer, is not guaranteed or endorsed by the publisher.

## References

[ref9001] Abdi H. (2010). Partial least squares regression and projection on latent structure regression. WIREs Comput. Stat. 2, 97–106. doi: 10.1002/wics.51

[ref1] Akita H. Matsuchika A. Kimura Z. I. (2019). *Enterobacter oligotrophica* sp. nov., a novel oligotroph isolated from leaf soil. Microbiologyopen 8:e843. doi: 10.1002/mbo3.843, PMID: 31066221 PMC7650607

[ref2] Aro J. M. Nyam-Osor P. Tsuji K. Shimada K. Fukushima M. Sekikawa M. (2010). The effect of starter cultures on proteolytic changes and amino acid content in fermented sausages. Food Chem. 119, 279–285. doi: 10.1016/j.foodchem.2009.06.025

[ref3] Banožić M. Jokić S. Ačkar Đ. Blažić M. Šubarić D. (2020). Carbohydrates-key players in tobacco aroma formation and quality determination. Molecules 25:1734. doi: 10.3390/molecules25071734, PMID: 32283792 PMC7181196

[ref4] Bastian M Heymann S Jacomy M (2009) Gephi: an open source software for exploring and manipulating networks. Proceedings of the third international conference on weblogs and social media, ICWSM 2009, San Jose, California, USA

[ref5] Caporaso J. G. Kuczynski J. Stombaugh J. Bittinger K. Bushman F. D. Costello E. K.. (2010). QIIME allows analysis of high-throughput community sequencing data. Nat. Methods 7, 335–336. doi: 10.1038/nmeth.f.303, PMID: 20383131 PMC3156573

[ref6] Chen J. Zheng Y. Guo Y. Li F. Xu D. Chao L.. (2021). Differences in microbial communities from Quaternary volcanic soils at different stages of development: evidence from late Pleistocene and Holocene volcanoes. Catena 201:105211. doi: 10.1016/j.catena.2021.105211

[ref7] Coenye T. (2014). “The family Burkholderiaceae” in The prokaryotes. eds. Rosenberg E. DeLong E. F. Lory S. Stackebrandt E. Thompson F. (Berlin, Heidelberg: Springer)

[ref8] Douglas G. M. Maffei V. J. Zaneveld J. Yurgel S. N. Brown J. R. Taylor C. M.. (2019). PICRUSt2: an improved and customizable approach for metagenome inference. BioRxiv:672295. doi: 10.1101/672295

[ref9] Du J. Zhang X. Wu G. Zhou R. Cui Y. Shi X. (2016). Studies on leaf surface microflora of cigar-wrapper during artificial fermentation. Curr. Biotechnol. 6, 188–192. doi: 10.3969/j.issn.2095-2341.2016.03.07

[ref10] Giacomo M. D. Paolino M. Silvestro D. Vigliotta G. Imperi F. Visca P.. (2007). Microbial community structure and dynamics of dark fire-cured tobacco fermentation. Appl. Environ. Microb. 73, 825–837. doi: 10.1128/AEM.02378-06, PMID: 17142368 PMC1800767

[ref11] Guo W. M. Zhang J. Liu Y. Jz S. Wei C. Y. Yue L.. (2009). Research on relationship between sensory quality and plastid pigment and polyphenol in flue-cured tobacco. Acta Tabacaria Sinica 29, 112–122. doi: 10.1016/S1874-8651(10)60084-1

[ref13] Jin A (1982) Cigar production technology. China Light Industry Press, Beijing

[ref14] Krzywinski M. Schein J. Birol İ. Connors J. Gascoyne R. Horsman D.. (2009). Circos: an information aesthetic for comparative genomics. Genome Res. 19, 1639–1645. doi: 10.1101/gr.092759.109, PMID: 19541911 PMC2752132

[ref15] Li H. Li X. Duan Y. Zhang K. Q. Yang J. (2010). Biotransformation of nicotine by microorganism: the case of *Pseudomonas* spp. Appl. Microbiol. Biotechnol. 86, 11–17. doi: 10.1007/s00253-009-2427-4, PMID: 20091027

[ref16] Li N. Zeng D. Dai Y. Li D. L. Wang C. G. Lei J. S.. (2009). Isolation and identification on cultivable microorganisms from cigar leaf surface. J. Anhui Agr. Sci. 37, 11857–11858.

[ref17] Liu F. Wu Z. Zhang X. Xi G. Zhao Z. Lai M.. (2021). Microbial community and metabolic function analysis of cigar tobacco leaves during fermentation. MicrobiologyOpen 10:e1171. doi: 10.1002/mbo3.1171, PMID: 33970539 PMC8483401

[ref18] Macris B. J. (1984). Production and characterization of cellulase and beta-glucosidase from a mutant of *Alternaria alternata*. Appl. Environ. Microbiol. 47, 560–565. doi: 10.1016/0141-4607(84)90089-1, PMID: 16346494 PMC239720

[ref19] Masai E. Katayama Y. Nishikawa S. Fukuda M. (1999). Characterization of *Sphingomonas paucimobilis* SYK-6 genes involved in degradation of lignin-related compounds. J. Ind. Microbiol. Biotechnol. 23, 364–373. doi: 10.1038/sj.jim.2900747, PMID: 11423957

[ref21] Parada A. E. Needham D. M. Fuhrman J. A. (2016). Every base matters: assessing small subunit rRNA primers for marine microbiomes with mock communities, time series and global field samples. Environ. Microbiol. 18, 1403–1414. doi: 10.1111/1462-2920.13023, PMID: 26271760

[ref22] Parks D. H. Tyson G. W. Hugenholtz P. Beiko R. G. (2014). STAMP: statistical analysis of taxonomic and functional profiles. Bioinformatics 30, 3123–3124. doi: 10.1093/bioinformatics/btu494, PMID: 25061070 PMC4609014

[ref23] Rai A. N. Söderbäck E. Bergman B. (2000). Cyanobacterium-plant symbioses. New Phytol. 147, 449–481. doi: 10.1046/j.1469-8137.2000.00720.x, PMID: 33862930

[ref24] Reid J. J. Gribbons M. F. Haley D. E. (1944). The fermentation of cigar-leaf tobacco as influenced by the addition of yeast. J. Agr. Res. 69, 373–381.

[ref25] Rognes T. Flouri T. Nichols B. Quince C. Mahé F. (2016). VSEARCH: a versatile open source tool for metagenomics. PeerJ 4:e2584. doi: 10.7717/peerj.2584, PMID: 27781170 PMC5075697

[ref26] Rossetto P. B. Dourado M. N. Quecine M. C. Andreote M. C. Araujo W. L. (2011). Specific plant induced biofilm formation in *Methylobacterium* species. Braz. J. Microbiol. 42, 878–883. doi: 10.1590/S1517-83822011000300006, PMID: 24031703 PMC3768801

[ref27] Schuster E. Dunn-Coleman N. Frisvad J. Dijck P. V. (2002). On the safety of *aspergillus Niger*-a review. Appl. Microbiol. Biot. 59, 426–435. doi: 10.1007/s00253-002-1032-6, PMID: 12172605

[ref28] Stal L. J. (2015). “Cyanobacteria, diversity and evolution of” in Encyclopedia of astrobiology. eds. Gargaud M. Irvine W. M. Amils R. Cleaves H. J. Pinti D. L. Quintanilla J. C.. (Berlin: Springer)

[ref29] Tabanelli G. Coloretti F. Chiavari C. Grazia L. Lanciotti R. Gardini F. (2012). Effects of starter cultures and fermentation climate on the properties of two types of typical Italian dry fermented sausages produced under industrial conditions. Food Control 26, 416–426. doi: 10.1016/j.foodcont.2012.01.049

[ref30] Tang H. Wang L. Meng X. Ma L. Ping X. (2009). Novel nicotine oxidoreductase-encoding gene involved in nicotine degradation by *Pseudomonas putida* strain S16. Appl. Environ. Microb. 75, 772–778. doi: 10.1128/AEM.02300-08, PMID: 19060159 PMC2632140

[ref31] Toharisman A. Sugianto Guntaryo. (2008). Java tabak cigar tobacco PTPN 10 Publishing Surabaya.

[ref32] Usyk M. Zolnik C. P. Patel H. Levi M. H. Burk R. D. (2017). Novel ITS1 fungal primers for characterization of the mycobiome. mSphere 2:e00488-17. doi: 10.1128/mSphere, PMID: 29242834 PMC5729218

[ref33] Wang R. (2003) Tobacco chemistry. China Agriculture Press, Beijing

[ref34] Wang X. (2013) Changes of the main chemical components and the quality characteristic of cigar-wrapper during curing and fermentation in Tongxiang of Zhejiang province. Henan, Henan agricultural University

[ref35] Wang M. Z. Yang G. Q. Wang Z. Yao Y. Lu Z. (2011). Nicotine degradation by two novel bacterial isolates of *Acinetobacter* sp. TW and *Sphingomonas* sp. TY and their responses in the presence of neonicotinoid insecticides. World J. Microb. Biotechnol. 27, 1633–1640. doi: 10.1007/s11274-010-0617-y

[ref36] Yan W. Huang S. Zhu G. Li Y. Huang F. Zhou X. (2018). Taxonomy and identification of microorganism causing mold damage of stored tobacco leaf in Guangxi. Tobacco Sci. Technol. 2, 50–56. doi: 10.3969/j.issn.1002-0861.2008.02.013

[ref37] Ye C. Li L. He C. Li D. Chen L. Fan L.. (2021). Structure and diversity analysis of microbial communities in cigar products by high-throughput sequencing technology. Tobacco Sci. Technol. 54, 1–9. doi: 10.16135/j.issn1002-0861.2021.0610

[ref38] Zhang Q. Geng Z. Li D. Ding Z. (2020a). Characterization and discrimination of microbial community and co-occurrence patterns in fresh and strong flavor style flue-cured tobacco leaves. MicrobiologyOpen 9:e965. doi: 10.1002/mbo3.965, PMID: 31808296 PMC7002102

[ref39] Zhang G. Li Z. Deng S. Li D. Zhang L. Cai B.. (2021). Characterization and succession analysis of bacterial community diversity in different fermentation cycles of Hainan H382 cigar leaf. Acta Tabacaria Sin. 27, 117–126. doi: 10.16472/j.chinatobacco.2020.170

[ref40] Zhang G. Liang K. Xin Y. Wang J. Li S. Wang F.. (2018a). Isolation and activity determination of surface bacteria in cigar wrapper leaves from four different countries. Chin. Tobacco Sci 39, 82–88. doi: 10.13496/j.issn.1007-5119.2018.02.012

[ref41] Zhang G. Liang K. Xin Y. Wang J. Liu H. (2018b). Diversity and succession of bacteria during the fermentation of a cigar wrapper using high throughput sequencing technology and traditional isolation. Chin. J. Appl. Environ. Biol. 4, 783–788. doi: 10.19675/j.cnki.1006-687x.2017.11014

[ref42] Zhang Q. Luo C. Li D. Cai W. (2020b). Research progress in curing and fermentation technology for cigar tobacco leaf production. Acta Tobacco Sin. 26, 1–6. doi: 10.16472/j.chinatobacco.2019.339

[ref43] Zhou Y. Bastida F. Zhou B. Sun Y. Gu T. Li S.. (2020). Soil fertility and crop production are fostered by micro-nano bubble irrigation with associated changes in soil bacterial community. Soil Biol. Biochem. 141:107663. doi: 10.1016/j.soilbio.2019.107663

